# Obstetrics and Gynæcology

**Published:** 1893-05

**Authors:** 


### *SELECTIONS AND ABSTRACTS.*

---

### OBSTETRICS AND GYNÆCOLOGY.

---

EDITED BY DR. VIRGIL O. HARDON.

---

### THE INFLUENCE OF ERGOT ON THE INVOLUTION OF THE UTERUS DURING THE LYING-IN PERIOD.

In the *Lancet* for November 19, 1892, Mr. Herman contributes the following article:

In the "Transactions of the Obstetrical Society of London," vol. xxx., for 1888, will be found a paper by Dr. C. Owen Fowler and Mr. Herman, in which observations are detailed pointing to this general conclusion: "That the administration of an ergot mixture during the first fortnight of the lying-in period appreciably increases the rapidity with which the diminution in size of the uterus goes on." This conclusion was reached by comparing the average rate of involution (*a*) in a number of cases, taken without selection, in which ergot was given, with (*b*) the average rate of involution in an equivalent number of cases, also taken without selection, in which ergot was not given. In the *Annales de Gynecologie*, vol. xxix., for 1888, p. 175, is published an investigation by Dr. Emile Blanc, of Lyons, conducted in a very similar way, but which led him to the conclusion that "ergotine administered during the first five or ten days of the lying-in period exerts no favorable influence on uterine involution." Dr. Blanc's research was quoted at the time in several English journals. These two investigations seem to contradict one another. Mr. Herman desires to point out that they do not; but that, on the contrary, they confirm one another, and show the real value of ergot in the lying-in period. The reason that Dr. Blanc's conclusion differs from that of Dr. Fowler and Mr. Herman is this, that he chose the cases in which to test the effect of ergot. He took only cases of "normal delivery at full term, excluding premature labors, cases with febrile disturbance, and all cases needing any intervention" (p. 177). These cases excluded are just those in which the causes known to hinder involution are present. Dr. Fowler and Mr. Herman took cases without any selection, and therefore among theirs were included cases in which the causes of subinvolution were present. Dr. Blanc's research shows that in a normal lying-in the uterus completes its involution as well without ergot as with it. The paper by Dr. Fowler and Mr. Herman shows the beneficial effect of ergot in counteracting the causes which retard involution. Dr. Blanc's paper contains nothing in opposition to this view; on the contrary, he expressly says, "Against secondary hemorrhage the drug maintains its position. Its action is the more efficacious the nearer the delivery." The practical conclusion is, that while in a perfectly normal lying-in ergot is not required, yet when any cause of imperfect involution is present, or suspected to be present, ergot, given throughout the lying-in period, will counteract its influence, will promote involution, and should be given.

---

### CERTAIN ASPECTS OF GONORRHŒA IN WOMEN.

Dr. Noble (*American Journal of Obstetrics*) says an interesting phase of gonorrhœa in women is the invasion of the womb, Fallopian tubes, ovaries and peritoneum. In the urethra, the vulvo-vaginal glands, the vagina, the uterus and the Fallopian tubes, the general facts are the same—the disease has little if any tendency to undergo a spontaneous cure. The rule is that a chronic catarrhal condition succeeds the acute inflammation—if the disease has not been chronic or "creeping" from the beginning—and that in some fold of membrane, crypt or follicle enough of the specific poison remains to set up acute inflammation anew. The known chronicity of the disease and its rebelliousness to treatment in accessible regions offer but little encouragement to expect a perfect cure in an inaccessible tube from which drainage is difficult, if not impossible. Personally, he knew of no case in which a gonorrhœal salpingitis had been perfectly cured. He believes that the rule of practice should be to remove all such uterine appendages when the health of the patient is compromised by their presence. There is reason to believe that gonorrhœal salpingitis invariably produces occlusion of the tube, except in those cases where the infection spreads quickly to the peritoneum and induces rapidly fatal peritonitis. In respect to the question as to removing both uterine appendages when only one is infected with gonorrhœa, he mentions the fact that when one uterine appendage has been removed for inflammation the disease is likely to attack the other tube subsequently. Therefore, in operating upon women the mothers of families, and who are approaching the menopause, it is certainly wise surgery to remove both uterine appendages, even though one is healthy. In young women desirous of bearing children, where only one tube is infected, it should be left to them to elect whether one or both tubes should be removed, as they alone must suffer the consequences of success or failure. Probably the percentage in which extension to the healthy side will occur can be materially reduced by appropriate treatment.—*Sheffield Medical Journal.*

---

### A NEW METHOD OF ARTIFICIAL RESPIRATION IN ASPHYXIA NEONATORUM.

Dew describes in the *Medical Record,* March 11, 1893, a procedure for resuscitating an asphyxiated infant, which is as follows: Grasp the infant with the left hand, allowing the neck to rest between the thumb and forefinger, the head falling far over backward; the mouth is straightened and also the larynx, the upper portion of the back and scapulæ resting in the palm of the hand; the other three fingers to be inserted in the axilla of the baby's left arm, raising it upward and outward. With the right hand the knees are so grasped that the right knee rests between the thumb and the forefinger, the left between the fore and middle fingers, this position allowing the back of the thighs to rest in the palm of the operator's hand. If the child is small, it will be found more convenient to grasp it by the ankles, allowing the calves, instead of the thighs, to rest in the palm of the hand. The next step is to depress the pelvis and lower extremities, so as to allow the abdominal organs to drag the diaphragm downward, with the left hand gently bending the dorsal region of the spine backward. This produces inspiration. The reverse of this movement, bringing the head, shoulders and chest forward, causes expiration. If the thighs are brought forward upon the abdomen, the lumbar region is arched backward and the child so bent upon itself as to produce a powerful expiration.—*American Journal of Medical Science.*

